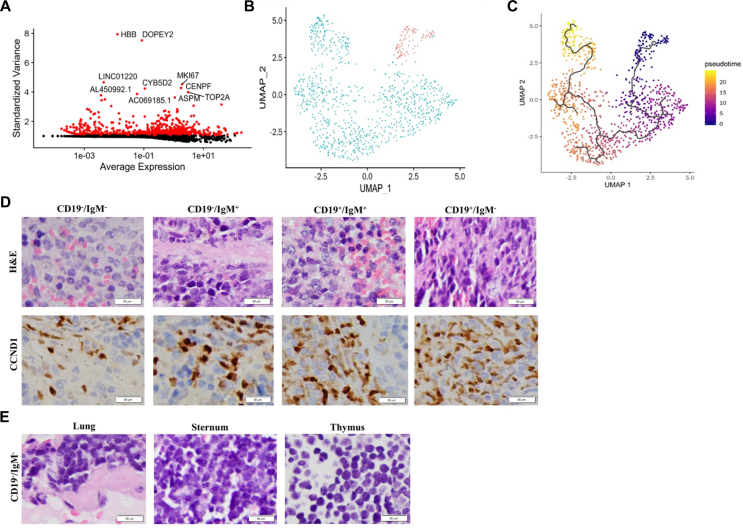# A novel research model of clonal evolution in mantle cell lymphoma at the single-cell genomic level

**DOI:** 10.1016/j.gendis.2024.101406

**Published:** 2024-09-01

**Authors:** Li Zhang, Yongsheng Liu, Liang Wang, Li Wang, Li Zheng, Wei He, Li Yan, Lvsu Ye, Huidan Zhang, Junling Tang

**Affiliations:** a Department of Occupational Disease and Poisoning Medicine, The First Affiliated Hospital of Chongqing Medical and Pharmaceutical College, Chongqing 400060, China; b Laboratory of Toxicology, The First Affiliated Hospital of Chongqing Medical and Pharmaceutical College, Chongqing 400060, China; c Chongqing Key Laboratory of Prevention and Treatment for Occupational Diseases and Poisoning, The First Affiliated Hospital of Chongqing Medical and Pharmaceutical College, Chongqing 400060, China; d Chongqing Medical and Pharmaceutical College, Chongqing 401331, China; e School of Engineering and Applied Sciences, Harvard University, Cambridge, MA 02138, USA; f Department of Hematology, Beijing Tongren Hospital, Capital Medical University, Beijing 100730, China

Mantle cell lymphoma (MCL) is recognized as one of the most genetically heterogeneous diseases, with high instability at the genomic level. MCL is common in males with a male-to-female ratio of about 2:1, and its incidence accounts for 3%–10% of adult non-Hodgkin lymphoma cases. The evolutionary dynamics of MCL clones at the single-cell level remain largely unclear. Our research suggests that MCL may arise from multiple cells within the abnormal microenvironment of the entire hematopoietic lineage, particularly from initiating cells. These initiating cells predominantly consist of CD19^−^/IgM^−^ subclones and exhibit a disrupted malignant clonal differentiation of pre-B cells along the tumor immunity evolution tree. Based on the single-cell transcriptome sequencing on the RedRock capture platform, we established the JeKo-1-LZ1 model, revealed the JeKo-1-LZ1 biology characteristics, and verified that the JeKo-1-LZ1 model was more representative than the JeKo-1-spheroid model by flow cytometry and sorting, colony forming experiment, and immunohistochemical and function analyses. Further details of our methodology can be found in the supplementary materials.

The accuracy and consistency of the RedRock system were confirmed, categorizing cells into nine distinct types (Fig. 1A; Fig. S1A). Additionally, utilizing cell annotation based on the atlas of blood cells (ABC) enabled the identification of all hematopoietic cell types^1^ (Fig. 1B). Subsequently, a JeKo-1-LZ1 model was established (Fig. 1C). The proportion of CD19^−^ initiating cells rose from 0.38% ± 0.11% in JeKo-1-parental cells to 79.23% ± 2.25% in regular spheroids, aligning with expression observed in clinical samples^2^^,^^3^ (Fig. 1D, E and Table S1). Cell viability decreased from 95.31% ± 0.48% in JeKo-1-parental cells to 65.48% ± 1.17% in irregular spheroids, but was restored to 94.78% ± 0.54% in regular spheroids (Fig. 1D, E and Table S1). Furthermore, the side population cells in regular spheroids accounted for 4.49% ± 0.05%, showing sensitivity to verapamil (50 μM) treatment (Fig. 1D). The JeKo-1-LZ1 cell line exhibited characteristics of a pure cell type with increased colony numbers (Fig. 1F; Fig. S1B), predominantly expressing IgM and κ light chain and weakly expressing IgD and κ light chain^2^ (Fig. S1C–G). Immunohistochemical analysis of the spleen (*n* = 4) and sternum (*n* = 2) revealed similar levels of CD79a, CD20, Pax5, IgM, CCND1, and Ki-67 (Fig. 1G and Table S2). Overall, JeKo-1-LZ1 displayed distinct features of leukemic nonmodal MCL biology, showing directional invasion towards the sternum without extensive tumor invasion.

**Figure 1:**
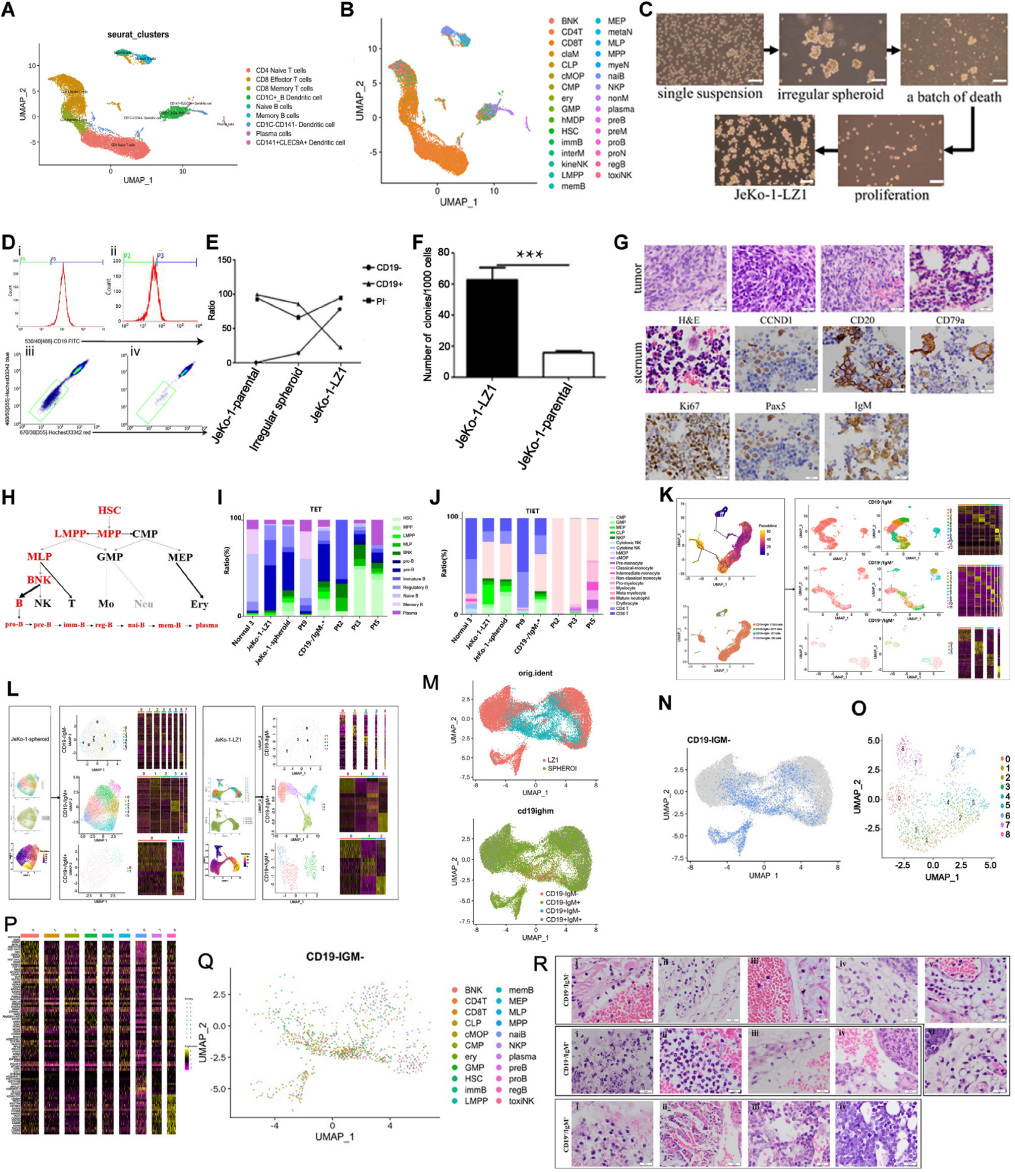
Single-cell transcriptomics sequencing reveals a new clonal evolution mechanism in mantle cell lymphoma. **(A)** Cell profile of normal 3 through manual annotation. In summary, normal 3 was composed of CD4 naïve T cells, CD8 effector T cells, CD8 memory T cells, CD1C^+^ B dendritic cells, naïve B cells, memory B cells, CD1C^−^CD14^−^ dendritic cells, plasma cells, and CD141^+^ECLC9A^+^ dendritic cells. **(B)** The annotation of normal 3 based on the atlas of blood cells (ABC). In summary, normal 3 was composed of BNK, CD4T, CD8T, claM, CLP, cMOP, CMP, ery, GMP, hMDP, HSC, immB, interM, kineNK, LMPP, memB, MEP, metaN, MLP, MPP, myeN, naiB, NKP, nonM, plasma, preB, preM, proB, proN, regB, and toxiNK. **(C)** A JeKo-1-LZ1 mode. The evolution of cell growth characteristics of JeKo-1-parental and JeKo-1-LZ1 cell lines. **(D)** The changes in the proportion of cells in clinical samples (pt2 and pt3). Figure D shows the expression of CD19^−^ in JeKo-1-parental cells (i) and JeKo-1-LZ1 cells (ii) and the expression of side population in JeKo-1-LZ1 cells incubated in Hoechst 33342 alone (iii) and Hoechst 33342 accumulation in the presence of 50 μM verapamil (iv). **(E)** The dynamics of phenotypic proportions of CD19^–^, CD19^+^, and PI^–^ in the three distinct growth modes. The proportion of CD19^+^ decreased first and then went up based on the order: JeKo-1-parental, Irregular spheroid, and JeKo-1-LZ1. The proportion of CD19^−^ decreased gradually based on the order: JeKo-1-parental, Irregular spheroid, and JeKo-1-LZ1. The proportion of PI^−^ increased gradually based on the order: JeKo-1-parental, Irregular spheroid, and JeKo-1-LZ1. **(F)** The quantitation of colony formation assay by JeKo-1-LZ1 and JeKo-1-parental. JekO-1-LZ1 was composed of many more cell clones. **(G)** The hematoxylin and eosin staining and immunohistochemical staining of JeKo-1-LZ1-derived tumor xenografts, including CCND1, CD20, CD79a, Ki67, Pax5, and IgM. Bar = 50 μm. **(H)** The evolution of differentiation from hematopoietic stem cells. In summary, from hematopoietic stem cell (HSC) to mature myeloid or lymphoid. **(I)** The cell proportions of the tumor evolution tree. In summary, the cell proportions of variation samples, including HSC, MPP, LMPP, MLP, BNK, pro-B, pre-B, immature B, regulatory B, naïve B, memory B, and plasma. **(J)** The proportions of the tumor immunity evolution tree. In summary, the cell proportions of variation samples, including CMP, GMP, MEP, CLP, NKP, cytotoxic NK, cytokine NK, hMDP, cMDP, pre-monocyte, classical-monocyte, intermediate-monocyte, non-classical monocyte, myelocyte, meta myelocyte, mature neutrophil, erythrocyte, CD4 T, and CD8 T. **(K)** The pseudotime analysis of normal 3 and the characteristics of the four subclones. CD19^−^/IgM^−^ is the main subclone and CD19^−^/IgM^+^ is the original subclone in normal 3. **(L)** The characteristics of the four subclones in JeKo-1-spheroid and JeKo-1-LZ1. CD19^−^/IgM^+^ is the main subclone in both JeKo-1-spheroid and JeKo-1-LZ1. **(M)** The expression of mixed samples from JeKo-1-LZ1 and JeKo-1-spheroid cells. CD19^−^/IgM^+^ subclone distributed widely in combined UMAP. **(N)** The CD19^−^/IgM^−^ subclone (blue) in the mixed sample. CD19^−^/IgM^−^ subclone is the second subclone in mixed UMAP. **(O)** The clustering analysis of the CD19^−^/IgM^−^ subclone in the mixed sample. There are 9 clusters after clustering the CD19^−^/IgM^−^ subclone. **(P)** The heatmap of the CD19^−^/IgM^−^ subclone in the mixed sample. The heatmap shows the differential expressed genes of 9 clusters in CD19^−^/IgM^−^ clone. **(Q)** The cell annotation by Scmap of CD19^−^/IgM^−^ subclone (blue) in the mixed sample. In summary, cell types of CD19^−^/IgM^−^ subclone including BNK, CD4 T, CD8 T, CLP, cMOP, CMP, ery, GMP, HSC, immB, LMPP, memB, MEP, MPP, naiB, NKP, plasma, reB, proB, regB, and toxiNK. **(R)** The hematoxylin and eosin staining of the infection and hemorrhage from mice inoculated with CD19^−^/IgM^−^, CD19^−^/IgM^+^, and CD19^+^/IgM^+^ from pt1. Bar = 50 μm.

JeKo-1-LZ1 cells were divided into six clusters, each showing high expression of CCND1 (Fig. S1H–J). By analyzing marker genes of B and T cells, we observed that B cells constituted a major proportion, with a minor representation of T cells (Fig. S2A, B). Subsequent classification revealed that the clusters primarily comprised pre-B and immature-B cells (Fig. S2A). Upon mapping to the ABC, the distinctiveness of each cell cluster became more apparent and well-defined. The purity of JeKo-1-spheroid was confirmed, leading to the segmentation of cells into eight clusters, with each cluster exhibiting high expression of CCND1 and IgM (Fig. S2C–G). Variations in the expression levels of marker genes associated with homing and stem cells were detected among the clusters (Fig. S2H–J), distinguishing them as pre-B cells, CD4 memory T cells, and CD4 naïve T cells (Fig. S2B). The cell differentiation process was illustrated based on the ABC, showing a progression from pre-B cells to plasma cells. Moreover, we observed the differentiation of B cells from hematopoietic stem cells (HSCs) to plasma cells, which is called the tumor evolution tree including LMPP > MEP > CMP > MLP > GMP > MPP > HSC4^4^. Meanwhile, the immune differentiation stage includes CMP to terminal stages of natural killer/T cells, monocytes, granulocytes, and erythroblasts, which is called the tumor immunity evolution tree including erythroblasts > T cells > natural killer cells > monocytes)^5^ (Fig. 1H–J; Fig. S2A, B).

ABC platform was used to map our clinical sample cell types. The major cell types were nucleated erythrocytes and other blood cells were also observed. Moreover, pt95 consisted of the above-mentioned cells (Fig. 1I, J; Fig. S3). Erythroblasts were associated with both innate and adaptive immune responses. The patients exhibited hemoglobin levels exceeding 100 g/L (Table S3), suggesting that abnormal erythroblast proliferation may stem more from immune dysfunction within the tumor immunity evolution tree rather than from anemia.

The functional analysis of JeKo-1-LZ1 and JeKo-1-spheroid highlighted their roles in cell cycle regulation, DNA and RNA synthesis, autoimmune diseases, and various neoplastic conditions such as lymphoma and leukemia (Fig. S4A, B). A similar analysis of clinical samples (pt2, pt3, and pt5) indicated a close association with immune function, autoimmune disorders, and hematopoietic system diseases (Fig. S4C–E). Notably, the protein CD74 was central in the network of JeKo-1-spheroid, pt5, and pt3 (Fig. S4B–D), suggesting that CD74^+^ erythroblasts with immune functionality may play a crucial role in the tumor immunity evolution tree^1^ (Fig. 1H–J; Fig. S2A, B, 4).

CD19 and IgM expression profiles varied across healthy, MCL, and chronic lymphocytic leukemia samples (Fig. 1K, L). In normal sample 3, cluster 6 was identified as the original clone (Fig. S5 and Table S4), comprising three subclones, namely in the descending order, CD19^−^/IgM^+^, CD19^+^/IgM^+^, and CD19^+^/IgM^−^ cells (Fig. S5A, B). CD19^−^/IgM^−^ subclonal cells were excluded due to their limited presence. In JeKo-1-spheroid and JeKo-1-LZ1, four subclones were observed, namely in the descending order, CD19^−^/IgM^−^, CD19^−^/IgM^+^, and CD19^+^/IgM^+^ cells (Fig. 1L and Table S5); the number of CD19^+^/IgM^−^ subclone cells was also low. Notably, CD19^−^/IgM^−^ cells were predominantly expressed in the initial clones of clusters 2/3 (JeKo-1-LZ1) and 0/4 (JeKo-1-spheroid) (Fig. 1J; Fig. S1H, 2D, 5C–F). Utilizing marker expression of CCND1, SOX11, CD79A, CD79B, and MS4A1, we further validated JeKo-1-LZ1 as a more representative model versus JeKo-1-spheroid (Fig. S6A, B).

The presence of the four subclones, including the CD19^−^/IgM^−^ subclone predominantly found in MCL, was confirmed in clinical samples (pt1–7) and pt9 (Fig. S6C and Table S4). To ascertain the differences in CD19 and IgM expression between MCL and chronic lymphocytic leukemia, analysis of chronic lymphocytic leukemia sample (pt8) revealed an abundance of the four subclones, namely in the descending order, CD19^−^/IgM^−^, CD19^+^/IgM^−^, CD19^−^/IgM^+^, CD19^+^/IgM^+^, and CD19^+^/IgM^−^ cells, compared with normal and MCL samples (Fig. S6D and Table S4).

MCL-initiating cells are likely predominantly composed of CD19^−^/IgM^−^ cells. By analyzing mixed cells from JeKo-1-LZ1 and JeKo-1-spheroid, specific cell types were identified as pre-B and immature-B cells. Mapping these cells to the ABC revealed the full spectrum of hematopoietic cells (Fig. 1M–Q). Ten feature genes showed significant cell-to-cell variation in the dataset, including HBB, DOPEY2, LINC01220, CYB5D2, MKI67, CENPF, AL 450992.1, AC06 9185.1, ASPM, and TOP2A (Fig. S7A). Notably, genes like HBB and CYB5D2, CENPF and TOP2A, and ASPM exhibited specific expression in erythroblasts, pre-B cells, and promonocytes, respectively. Upon further mapping to ABC, CD19^−^/IgM^−^ cells from the mixed sample were divided into nine clusters, with the presence of hematopoietic stem progenitors, B cells, erythroblasts, T cells, and natural killer cells (Fig. 1I, J, M–Q). JeKo-1-LZ1 cells were observed to be localized at the earliest stage, as depicted in the xenotransplantation assay showing their ability to spread to the sternum (Fig. 1G; Fig. S7B, C and Table S2).

*In vivo*, CD19^−^/IgM^−^ cells displayed the most aggressive tumor behavior. Four subclones isolated from tumor masses formed in the spleen of patient pt1 exhibited retained high CCND1 expression (Fig. S7D), while CD20, CD79a, PAX 5, Ki-67, and IgM expression were not detected (data were not shown). However, CD19^−^/IgM^−^ cells demonstrated invasive properties towards distant organs like the sternum, lung, and thymus (Fig. S7E), suggesting stronger tumor invasion capabilities versus other subclones. Only groups showing pathological signs of infection and hemorrhage were the CD19^−^/IgM^−^, CD19^−^/IgM^+^, and CD19^+^/IgM^+^, with the CD19^−^/IgM^−^ subclone being the most predominant among them (Fig. 1R and Table S6). These findings further support the CD19^−^/IgM^−^ subclone as the potential tumor-originating clone.

## Ethics declaration

All sample collection procedure was approved by the Ethics Committee of The Affiliated Hospital of Southwest Medical University (KY2019091) and written informed consent was obtained from patients based on the Declaration of Helsinki.

## Author contributions

**Li Zhang:** Data curation, Formal analysis, Writing – original draft. **Yongsheng Liu:** Data curation, Formal analysis. **Liang Wang:** Data curation, Formal analysis. **Li Wang:** Project administration. **Li Zheng:** Formal analysis. **Wei He:** Project administration. **Li Yan:** Project administration. **Lvsu Ye:** Project administration. **Huidan Zhang:** Data curation, Formal analysis. **Junling Tang:** Project administration, Supervision.

## Conflict of interests

The authors declared no potential conflict of interests.

## Funding

This work was funded by the Chongqing Medical Scientific Research Project (Joint Project of Chongqing Health Commission and Science and Technology Bureau, China) (No. 2023GGXM006, 2024ZDXM026, 2024MSXM115), the Key Research Project from Science and Technology Department of Sichuan Province, China (No. 2019YFS0301), the Chongqing Key Municipal Public Health Specialty Construction Project (China), the Key Research Project from Chongqing Medical and Pharmaceutical Vocational Education Group (China) (No. CQZJ202329), the Incubation Project of the First Affiliated Hospital of Chongqing Medical and Pharmaceutical College (China) (No. 2022-2023ZD04, 2022-2023ZD03, 2022-2023MS04, 2022-2023MS03, 2022-2023MS010), and the Science and Technology Research Program of Chongqing Education Commission (China) (No. KJQN202302811).

## Data availability

Most data used in this research have been uploaded as files or supplementary files and raw sequence data will be supplied for reasonable requirements through contacting the corresponding author.

## Figures and Tables

**Figure:**
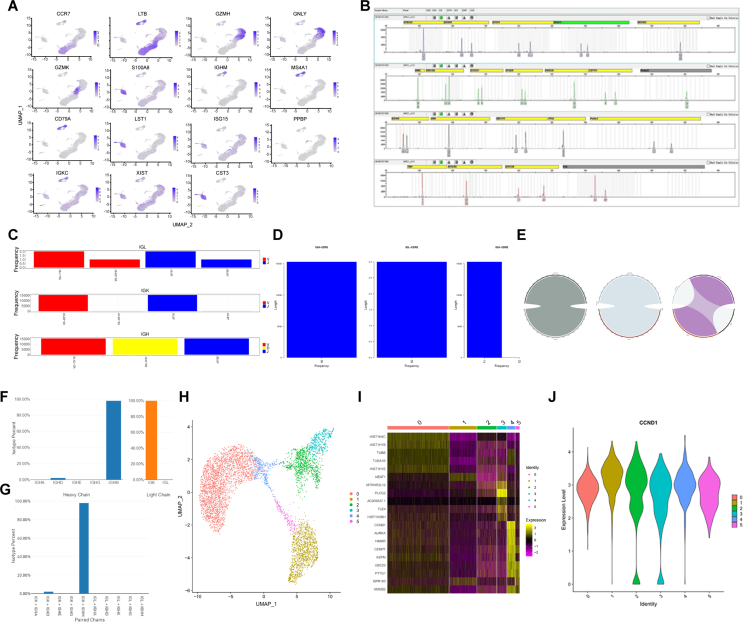
figs1

**figs2:**
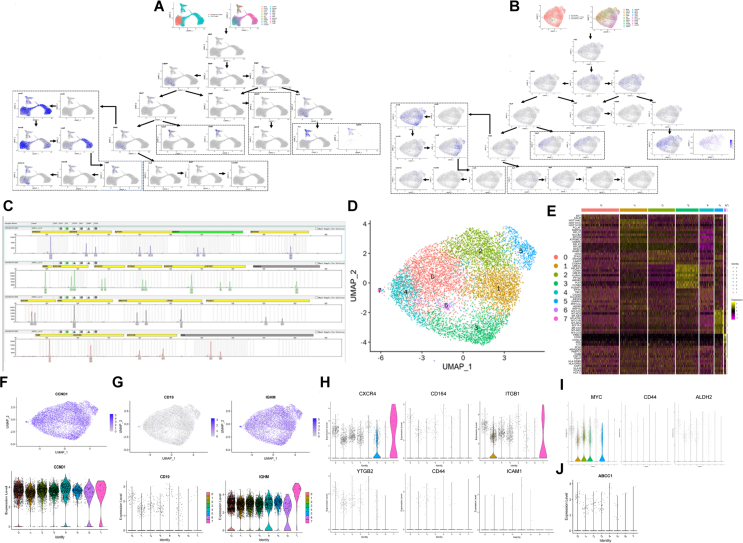


**figs3:**
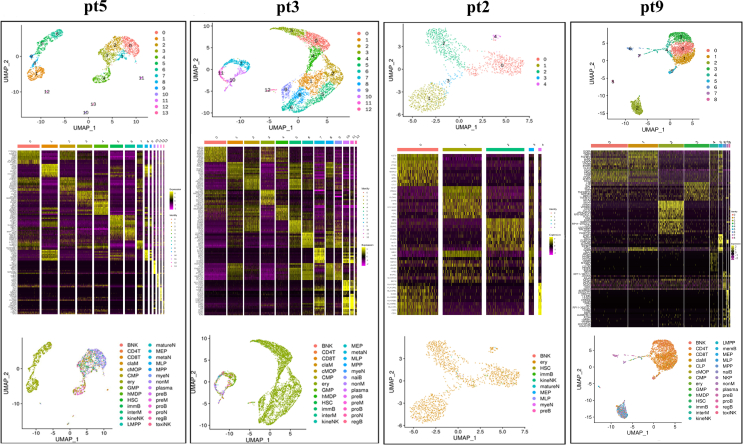


**figs4:**
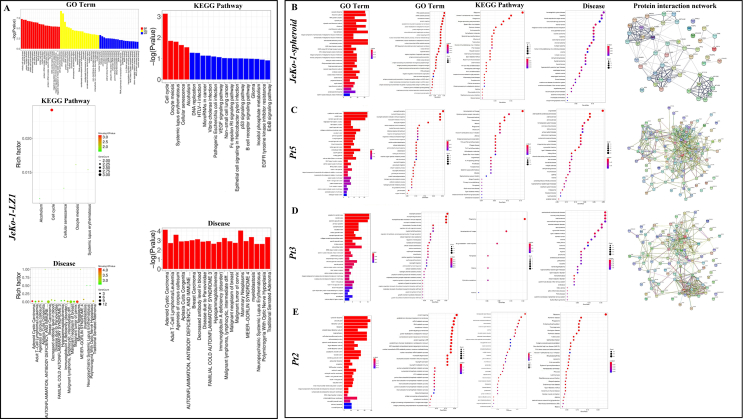


**figs5:**
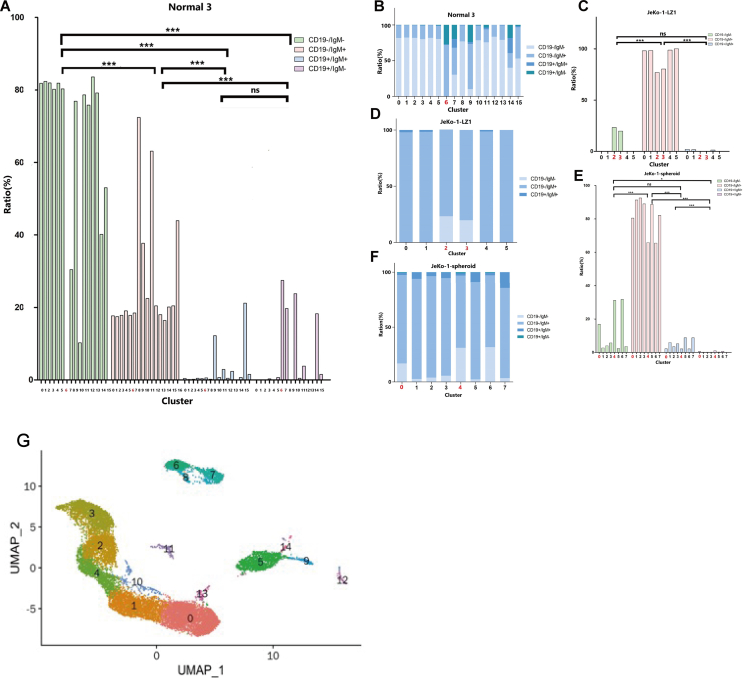


**figs6:**
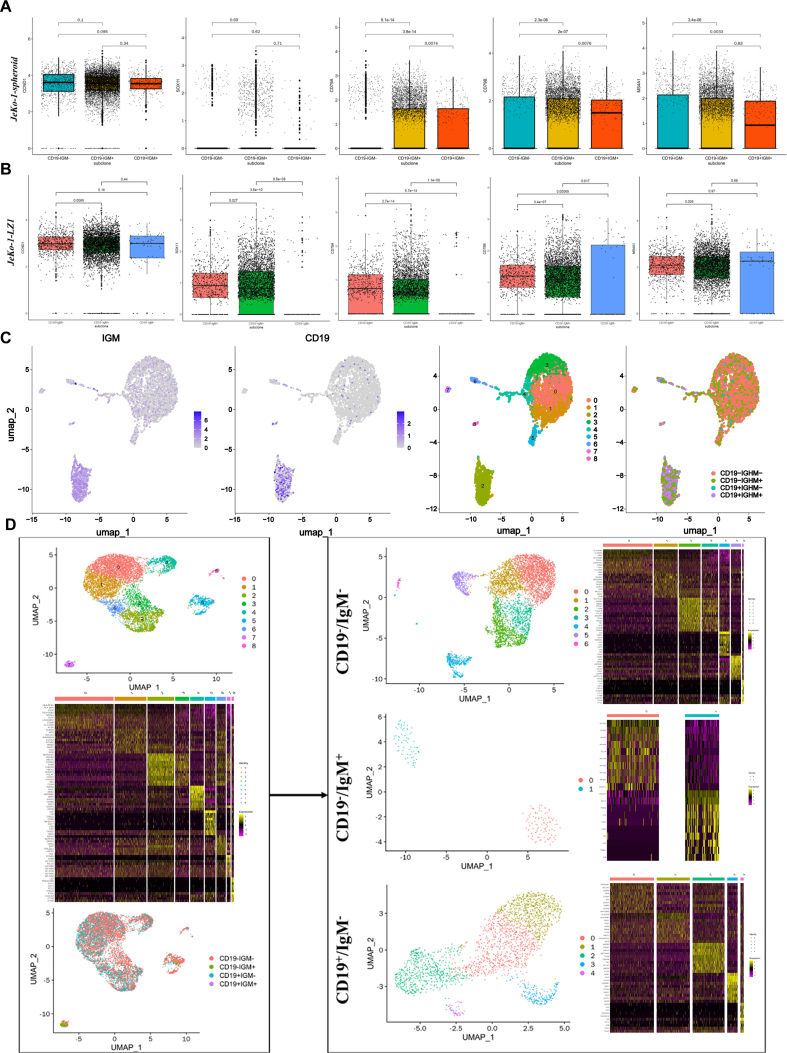


**figs7:**