# Supplementary material for: A novel research model of clonal evolution in mantle cell lymphoma at the single-cell genomic level
Source: Genes Dis. 2024 Sep 1;12(3):101406. doi: 10.1016/j.gendis.2024.101406 (PMC11795050; doi:10.1016/j.gendis.2024.101406)
Supplement: Multimedia component 1 [file mmc1.docx]

**Supplementary figure legend**

**Supplementary Fig. 1 Establishment of the *JeKo-1-LZ1* cell line.**

**(A)** Expression patterns of selected markers projected on the UMAP plot. **(B)** The STR test of *JeKo-1-LZ1* cells. **(C)** Frequency histograms of V(D)J genes. **(D)** The length profile of the CDR3 amino acid sequence. **(E)** The frequency map of V(D)J genes. **(F)** Proportion of heavy and light chains. **(G)** Proportion of paired chains. **(H)** Clustering analysis of *JeKo-1-LZ1* cells. **(I)** Heatmap of *JeKo-1-LZ1* cells. **（J）**The expression level of CCND1 in clusters.

**Supplementary Fig. 2** **Characteristics of the *JeKo-1-LZ1 and JeKo-1-spheroid cell lines.***

**(A)** Atlas of *JeKo-1-LZ1*. **(B)** Atlas of *JeKo-1-spheroid*. **(C)** The STR test of *JeKo-1-**spheroid* cells. **(D)** Clustering analysis of *JeKo-1-spheroid* cells. **(E)** Heatmap of *JeKo-1-spheroid* cells. **(F)E**xpression level of CCND1 in clusters. **(G)** Expression level of CD19 and IgM. **(H)** Expression level of homing genes. **(I)** Expression level of stem cell genes. **(J)** Expression level of ABCC1.

**Supplementary Fig. 3 Cell type of the clinical samples.**

**Supplementary Fig. 4 Functional analysis of** ***JeKo-1-LZ1*,** ***JeKo-1-spheroid*, pt2, pt3, and** **pt5.**

**(A)** Functional analysis of *JeKo-1-LZ1*. **(B)** Functional analysis of *JeKo-1-spheroid*. **(C)** Functional analysis of pt5. **(D)** Functional analysis of pt3. **(E)** Functional analysis of pt2.

**Supplementary Fig. 5 Expression of CD19^−^/IgM^−^, CD19^−^/IgM^+^, CD19^+^/IgM^+^, and CD19^+^/IgM^−^ subclones in the different clusters (the red tag is the initial clone).**

**Supplementary Fig. 6 Expression of CD19^−^/IgM^−^, CD19^−^/IgM^+^, CD19^+^/IgM^+^, and CD19^+^/IgM^−^ subclones at scRNA-seq level.**

(A) Expression levels of CCND1, SOX11, CD79A, CD79B, and MS4A1 in the CD19^−^/IgM^−^, CD19^−^/IgM^+^, and CD19^+^/IgM^+^ subclones of *JeKo-1-spheroid*. (B) Expression levels of CCND1, SOX11, CD79A, CD79B, and MS4A1 in the CD19^−^/IgM^−^, CD19^−^/IgM^+^, and CD19^+^/IgM^+^ subclones of *JeKo-1-LZ1*.(C) The expression of CD19 and IgM in pt9. (D) The clustering analysis and the expression of CD19 and IgM of pt8.

**Supplementary Fig. 7 Cancer biology of** **CD19^−^/IgM^−^, CD19^−^/IgM^+^, CD19^+^/IgM^+^, and CD19^+^/IgM^−^ subclones.**

**(A)** The 10 characteristic genes with high variation in the mixed sample of *JeKo-1-spheroid* and *JeKo-1-LZ1*; red indicates genes with significant differences. **(B)** The UMAP of JeKo-1-LZ1 (red) and JeKo-1-spheroid (green) in the CD19^−^/IgM^−^ subclone. **(C)** Pseudotime analysis of the CD19^−^/IgM^−^ subclone. **(D)** The H&E staining and CCND1 IHC of the four subclones from pt1 in the spleen. **(E)** Morphological features of the CD19^−^/IgM^−^-derived tumor xenografts from pt1.
