# Supplementary material for: A novel research model of clonal evolution in mantle cell lymphoma at the single-cell genomic level
Source: Genes Dis. 2024 Sep 1;12(3):101406. doi: 10.1016/j.gendis.2024.101406 (PMC11795050; doi:10.1016/j.gendis.2024.101406)
Supplement: Multimedia component 2 [file mmc2.docx]

**Supplementary Material and Methods**

**Methods**

**Cell line and patient sample**

*JeKo-1-LZ1* cells maintained in RPMI 1640 (HyClone) supplemented with 2% B27 (Excell). *JeKo-1-parental* and *JeKo-1-spheroid* cells were cultured following the description of a previous experiment.^4^ Specimens from patients and healthy donors were obtained following informed consent, as approved by Institutional Review Boards. To enrich the dominant clones, MCs were isolated and cultured for three generations, following the method in a previous experiment.^1^

**Side population (SP) assay**

The Hoechst 33342 staining procedure was based on the method by Goodell et al.^1^ and observed using Becton–Dickinson Aria III.

**Flow cytometry and sorting**

Cells were incubated following the method described in a previous experiment and analyzed using Becton–Dickinson Aria III. The following were the antibodies: IgM-APC (catalog: 551062, BD Biosciences), CD19-FITC (catalog: 11-0199, eBioscience), and propidium iodide (PI, Sigma). The following were the gating strategies: gate I, PI^–^ and gate ii, CD19^–^/IgM^–^; CD19^–^/IgM^+^, CD19^+^/IgM^+^, and CD19^+^/IgM^–^. The sorting purity was greater than 99%.

**STR test**

Cells were sent to China Center for Type Culture Collection for STR profiling identification.

**Colony formation assay**

Cells were plated 1,000 cells per well, following the method described in a previous experiment.^1^

**Xenotransplantation and immunohistochemistry (IHC) of NOD/SCID mice**

All mice were obtained from the core facility of the Experimental Animal Centre, as approved by the Animal Care Committee. *JeKo-1-LZ1* cells were intraperitoneally injected at doses of 10^6^ cells per NOD/SCID mouse (n = 4, 21 days). CD19^−^/IgM^−^, CD19^−^/IgM^+^, CD19^+^/IgM^+^, and CD19^+^/IgM^−^ cells from pt1 were intraperitoneally injected at two doses, 10^2^ and 10^4^ (n = 4, 10 days), respectively. The organs were collected for hematoxylin and eosin staining, and anti-human IgM (catalog: ab17104, Abcam), CD20 (catalog: Kit-0001, Maxim), CD79a (catalog: RMA-0552, Maxim), Ki-67 (catalog: ZA-0502, ZSGB), CCND1 (catalog: RMA-0541, Maxim), and Pax5 (catalog: AM0281, Ascend) were used for IHC, following the method described in a previous experiment.^1^

**RedRock: the novel single-cell capture platform**

Cell quality control was performed using the Countstar Fluorescence Cell Analyzer. The following were the cell requirements: viability ≥ 80%; diameter < 40 µm; concentration, 700–1,200 cells/µL; and total count, 1.0 × 10^5^ cells/sample.

**1 Single-cell isolation**

The cell, barcoding hydrogel beads, carrier oil, and RT–lysis mix were added into the channel of the microfluidic chip (**Figures S1A–C**); oil-in-water droplets were prepared on the RedRock platform (**Figure 1D**); and the droplets were collected into the centrifugal tube (**Figure S1C**).

**2 Cell barcode addition**

Each barcoding bead contained a large number of oligonucleotide sequences, which were composed of PCR primer, cell barcode, UMI, and poly dT (**Figure S1E**). In the droplet, the poly dT sequence was complementary to the mRNA polyA released after cell lysis to reverse transcription and synthesize cDNA (**Figure S1E**). The collection tube of oil-in-water droplets was put into the PCR instrument, and the temperature conditions of reverse transcription were as follows: reaction temperature, 42℃; reaction time, 90 min.

**3 Library construction**

Three milliliters of 1H,1H,2H, 2h-perfluorooctanol were added into the collection tube; blown 15 times; centrifuged (800 g, 10 min, 4℃); and collected the supernatant, which contains the RT product, namely cDNA. The RT product was purified by Dynabeads® MyOne™ SILANE, and the purified product was pre-amplified by PCR: 2× KAPA HIFI HotStart Ready Mix, 25 µL; purified reverse transcription product, 24 µL; pre-amplified primer, 1 µL. The PCR procedure is presented in **Table S1**. 0.6× KAPA Pure Beads were used for PCR amplification products, and LabChip GX Touch 24 Fluid C was used for detection. The detection results are shown in **Figure S1F**.

**4 Fragmented processing**

**4.1 Tn5 transposase was used for library construction**

Reaction system: 5× Tagment Buffer, 10 µL; pre-amplification product, 50 ng; Tn5 transposase, 5 µL; and supplemented with water to 50 µL. Reaction procedure: hot lid, 75℃, 55℃ 5 min, and 12℃ hold; after the reaction, 10-µL of 0.1% SDS solution was added to stop the reaction, blown and mixed, and incubated for 5 min at 25℃.

**4.2 The segmented products were purified with 1.0× KAPA Pure Beads.**

**4.3 Adding joint**

Reaction system: 2× PCR mix; fragment product, N5 joint (10 uM), N7 joint (10 uM); and added water to 50 µL.

**4.4 Reaction process as presented in Table S2**

**4.5 Product purification**

After purification of the library with 0.6× + 0.15× KAPA Pure Beads, the main peak was approximately 460 bp and detected by LabChip GX Touch 24 Fluid C.

**4.6 Sequencing**

After the library construction was completed, sequencing was performed on Illumina NovaSeq 6000, and the sequencing strategy was PE150 mode.

**5 Structure information of primers involved**

**5.1 Barcode structure**

**5.2 Primer used for reverse transcription**

**5.3 Primers used for pre-amplification**

**Single-cell RNA** **and BCR-V(D)J-****library construction and sequencing**

The study flowchart is presented in **Figure 1A**. The library preparation of single-cell RNA sequencing and single-cell BCR sequencing was performed using 10× Genomics and RedRock (Geno-Truth Biotechnology Ltd., Suzhou, China), according to the manufacturer’s protocol of the Single-Cell 5’ library construction and V(D)J enrichment. Barcoded sequencing libraries were conducted following Illumin’s protocol of the Novaseq 6000 platform. Paired-end high-throughput sequencing with 150-nt reads length was performed on NovaSeq 6000. Sequencing reads of one lane were generated for each sample. Reads alignment, feature-barcode matrices generation, sample clustering, cell type annotation, and other secondary analyses were performed using the Cell Ranger pipeline, manual annotation and the Atlas of Human Blood Cells database. The obtained GEPs were subjected to gene ontology and KEGG analysis using the R package clusterProfiler (v3.11). The atlas consists of cells directly involved in the differentiation of B cells from HSC to plasma cells, which is called the tumor evolution tree (TET). Meanwhile, the immune differentiation stage includes CMP to terminal stages of NK/T cells, monocytes, granulocytes, and erythroblast, which is called the tumor immunity evolution tree (TIET).^[1]^

A total of 125,379 cells were sequenced (3,989–63,124/sample) with a mean reads per cell of 57,964 reads. Experimental procedures followed established techniques using the Chromium Single-Cell 3’ Library V3 kit. Briefly, MCs were loaded into the 10× Genomics scRNA-seq: *JeKo-1-LZ1* (5,220 cells; 88,514 reads), pt9 (3,989 cells; 50,000 reads);^6^ 10× Genomics scBCR-V(D)J-seq: *JeKo-1-LZ1* (63,124 cells; 6,413 reads); RedRock scRNA-seq: *JeKo-1-spheroid* (8,992 cells; 94,693 reads), normal 3 (17,572 cells; 24,539 reads), pt2 (7,836 cells; 77,705 reads), pt3 (6,827 cells; 46,256 reads), pt5 (4,327 cells; 78,413 reads), and pt8 (7,492 cells; 55,141 reads).

**Statistical analysis**

Student’s t-test with Welch’s correction was used for the comparison between the two groups. By default, two-tailed tests were performed. ^*^: P < 0.05 was considered to be statistically significant. ^**^: P < 0.001,^***^: P <.0001. All analyses were performed using GraphPad Prism version 5.0.

**References:**

1.Junling Tang,Li Zhang,Tiejun Zhou,et al. Identification and characterization of the cellular subclones that contribute to the pathogenesis of mantle cell lymphoma.Genes Dis. 2019 Dec; 6(4): 407–418.
