# Supplementary material for: A novel research model of clonal evolution in mantle cell lymphoma at the single-cell genomic level
Source: Genes Dis. 2024 Sep 1;12(3):101406. doi: 10.1016/j.gendis.2024.101406 (PMC11795050; doi:10.1016/j.gendis.2024.101406)
Supplement: Multimedia component 3 [file mmc3.docx]

**Supplementary Table 1 The ratio of PI^-^, CD19^-^, CD19^+^ cells in three growth mode.**

| **Growth mode** | **PI** | **CD19^-^** | **CD19^+^** |
| --- | --- | --- | --- |
| *JeKo-1-parenta*l 95.31%±0.48% 0.38%±0.11% 99.62%±0.10%  Irregular spheroid 65.48%±1.17% 14.03%±0.85% 85.97%±0.85%  *JeKo-1-LZ1* 94.78%±0.54% 79.23%±2.25% 20.77%±2.25% | | | |

**Supplementary Table 2 The analysis of tumor xenografts of *JeKo-1-LZ1.***

| **Cell** | **# of mice with tumors in spleen** | **# of tumor-infiltrated sternum** | **# of tumor-infiltrated other organs*** | **# of infected organs** | **# of hemorrhagic organs** |
| --- | --- | --- | --- | --- | --- |
| ***JeKo-1-LZ1*  4/4 2/4 0/36 0/44 0/44** | | | | | |

***: Other organs include brain, thymus, heart, lung, liver, kidney and adrenal gland, stomach, intestines and pancreas.**

**Supplementary Table 3 The information of clinical samples.**

| **Patient**  **ID** | **Stage** | **WBC**  ×10^9^/L | **NEU**  ×10^9^/L | **LYM**  ×10^9^/L | **Hb**  g/L | **PLT**  ×10^9^/L | **IG%** | **LDH**  U/L | **UA**  umol/L | **Β2-MG**  mg/L | **IgM**  g/L |
| --- | --- | --- | --- | --- | --- | --- | --- | --- | --- | --- | --- |
| **1 IVB 67.12 5.17 30.94 56 37 42.7 NA NA NA 0.14**  **2 IVB 8.89 6.03 1.53 124 185 0 205.1 517.5 2.641 1.65**  **3 IVA 6.16 3.65 2.00 129 159 0 171.8 316.4 2.671 NA**  **4 IVB 11.55 7.51 2.64 99 276 0 234 560.3 NA NA**  **5 IVB 14.60 4.39 8.45 111 94 0 442.8 471.3 NA 2.56**  **6 IVA 10.26 6.36 3.04 92 8 0 478.1 NA NA NA**  **7 IVB 112.27 10.33 59.5 125 123 37 344.8 361.4 4.35 0.51**  **8 IV^*^ 46.63 5.60 39.64 127 106 1 208.6 374.1 NA NA** | | | | | | | | | | | |

^*^：Rai clinical staging system of CLL

Normal range:WBC(3.5-9.5×10^9^/L),NEU(1.8-6.3×10^9^/L),LYM(1.1-3.2×10^9^/L),Hb:130-175g/l,PLT:100-300×10^9^/L,IG%（0）,LDH（120-250U/L）,UA（208-428umol/L）,β2-MG（1.3-3.0mg/L）,IgM（0.4-2.8g/L）

**Supplementary Table 4. Top differential expressed genes of subclone in JeKo-1-spheroid, JeKo-1-LZ1 and Normal 3.**

**Part 1: JeKo-1-spheroid**

| **CD19^-^/IgM^-^** | |
| --- | --- |
| From top to bottom | **Features** |
|  | IGKC |
|  | ACTB |
|  | HERPUD1 |
|  | YBX1 |
|  | SRSF9 |
|  | HIST1H4C |
|  | HIST1H1C |
|  | HIST1H1E |
|  | HLA-DRB5 |
|  | HIST1H1D |
|  | NME2 |
|  | ID2 |
|  | MYBL2 |
|  | BRI3 |
|  | NUDT8 |
|  | HSPE1 |
|  | RPP25 |
|  | LAMTOR5 |
|  | ARL3 |
|  | TXN2 |
|  | POLD4 |
|  | POLR3K |
|  | CHURC1 |
|  | IGHG3 |
|  | HNRNPH1 |
|  | CALR |
|  | NUCKS1 |
|  | PTRH1 |
|  | CCR7 |
|  | STRBP |
|  | CTBP1 |
|  | APOBEC3C |
|  | RP1A |
|  | CLNS1A |
|  | CD74 |
|  | SLC25A5 |
|  | COPS6 |
|  | RANBP1 |
|  | HMGN2 |
|  | ADRM1 |
|  | GHITM |
|  | RPLP2 |
|  | RPL27A |
|  | RPS15A |
|  | RPL39 |
|  | RPS13 |
|  | RPS29 |
|  | RPS21 |
|  | RPL37 |
|  | RAMP1 |
|  | RPL30 |
|  | ARL6IP1 |
|  | AURKA |
|  | UBE2S |
|  | NUSAP1 |
|  | PTTG1 |
|  | CCNB2 |
|  | PLK1 |
|  | UBE2C |
|  | CCNB1 |
|  | CEEF1A1 |
|  | EZH2 |
|  | USP5 |
|  | PRRC2A |
|  | RIOK1 |
|  | ADNAJA1 |
|  | CEIPR1 |
|  | IMP4 |

| **CD19^+^/IgM^+^** | |
| --- | --- |
| From top to bottom | **Features** |
|  | RPL37 |
|  | COX17 |
|  | NDUFB3 |
|  | POLR2L |
|  | SRI |
|  | SNRPG |
|  | NDUFB1 |
|  | COX8A |
|  | HIST1H4C |
|  | CD19 |
|  | MT-CO1 |
|  | IGKC |
|  | CD74 |
|  | ENO1 |
|  | MT-CO3 |
|  | HLA-DRA |
|  | ATP5F1A |

| **CD19^-^/IgM^+^** | |
| --- | --- |
| From top to bottom | **Features** |
|  | SET |
|  | HIST1H4C |
|  | HIST1H1C |
|  | HIST1H1D |
|  | PCLAF |
|  | SNRPG |
|  | HIST1H1E |
|  | NUCB2 |
|  | POLR2L |
|  | ATP5MC1 |
|  | SNRPE |
|  | RPL39 |
|  | RPS29 |
|  | RAMP1 |
|  | CD52 |
|  | CD7 |
|  | LGALS1 |
|  | SNHG8 |
|  | S100A4 |
|  | IGHG3 |
|  | UBE2C |
|  | ARL6IP1 |
|  | CCNB1 |
|  | UBES2 |
|  | PTTG1 |
|  | AURKA |
|  | PLK1 |
|  | CCNB2 |
|  | NUSAP1 |
|  | CENPE |
|  | CD74 |
|  | EIF5A |
|  | MT-CO1 |
|  | YBX1 |
|  | PFN1 |
|  | CALM2 |
|  | SLC25A5 |
|  | PSAP |
|  | SNRPA1 |
|  | BUD23 |
|  | MT-CO2 |
|  | MT-ND1 |
|  | MT-ATP6 |
|  | MT-ND4 |
|  | MT-ND3 |
|  | MT-CYB |
|  | EEF1A1 |
|  | MT-ND2 |
|  | RPS20 |

**Part 2: Jeko-1-LY1**

| **CD19^-^/IgM^-^** | |
| --- | --- |
| From top to bottom | **Features** |
|  | PRD1 |
|  | CNBP |
|  | GSPT1 |
|  | GLRX3 |
|  | PSMD8 |
|  | LMAN2 |
|  | BUB1 |
|  | KIF22 |
|  | NDUFS2 |
|  | RALY |
|  | XIST |
|  | MT-CO2 |
|  | MT-ND3 |
|  | BOD1L1 |
|  | KCNQ1OT1 |
|  | OR6C75 |
|  | MT-ND2 |
|  | GOLGA4 |
|  | ANKRD11 |
|  | TARSL2 |
|  | PLCG2 |
|  | AC005837.1 |
|  | SNHG9 |
|  | RHOQ |
|  | SOX4 |
|  | ISL2 |
|  | C21orf58 |
|  | AC025164.1 |
|  | POLD2 |
|  | CAPN10-DT |
|  | SPRY1 |
|  | PNPLA4 |
|  | CUTA |
|  | HDDC3 |
|  | H2AFJ |
|  | ARHGAP21 |
|  | C15orf61 |
|  | C2orf49 |
|  | DHX9 |
|  | TCP1 |
|  | SCLY |
|  | MRS2 |
|  | OPA1 |
|  | HIPK2 |
|  | SS18L2 |
|  | UBP1 |
|  | ATG2A |
|  | CCNL1 |
|  | TTF1 |
|  | RBM20 |

| **CD19^+^/IgM^+^** | |
| --- | --- |
| From top to bottom | **Features** |
|  | HIST1H4C |
|  | CDT1 |
|  | HIST1H1B |
|  | HIST1H1E |
|  | PLCG2 |
|  | NSUN6 |
|  | SNHG25 |
|  | MTRNR2L12 |
|  | MTRNR2L12 |
|  | TLE4 |
|  | TOP2A |
|  | CCNB1 |
|  | ASPM |
|  | MKI87 |
|  | CENPF |

| **CD19^-^/IgM^+^** | |
| --- | --- |
| From top to bottom | **Features** |
|  | HIST1H4C |
|  | HIST1H1B |
|  | CDT1 |
|  | HIST1H1E |
|  | HIST2H2AC |
|  | CD19 |
|  | GABPB1-AS1 |
|  | NEAT1 |
|  | PLCG2 |
|  | KCNQ1OT1 |
|  | HMGB2 |
|  | TOP2A |
|  | CENPF |
|  | UBE2C |
|  | MKI87 |

**Part 3: normal 3**

| **CD19^-^/IgM^-^** | |
| --- | --- |
| From top to bottom | features |
|  | LTB |
|  | LDHB |
|  | GPR183 |
|  | AQP3 |
|  | TRAC |
|  | IL7R |
|  | MAL |
|  | TRADD |
|  | JUNB |
|  | CORQ1B |
|  | CCR7 |
|  | RPS13 |
|  | RPS3A |
|  | LEF1 |
|  | TCF7 |
|  | NOSIP |
|  | RCAN3 |
|  | TRADB2A |
|  | IL6ST |
|  | GZMH |
|  | NKG7 |
|  | FGFBP2 |
|  | CCL5 |
|  | KLRG1 |
|  | TRGC2 |
|  | GZMA |
|  | CST7 |
|  | PLEK |
|  | CD8A |
|  | GNLY |
|  | GZMB |
|  | KLRF1 |
|  | PRF1 |
|  | MYOM2 |
|  | TRDC |
|  | KLRD1 |
|  | SPON2 |
|  | CD8B |
|  | LINC02446 |
|  | LRRN3 |
|  | NELL2 |
|  | PDE3B |
|  | NUCB2 |
|  | PIK3IP1 |
|  | GZMK |
|  | DUSP2 |
|  | LYAR |
|  | CXCR4 |
|  | JUN |
|  | CD69 |
|  | CMC1 |
|  | TRGC1 |
|  | S100A8 |
|  | S100A9 |
|  | LYZ |
|  | VCAN |
|  | MNDA |
|  | FOS |
|  | CTSS |
|  | FCN1 |
|  | S100A12 |
|  | CST3 |
|  | CD79A |
|  | MS4A1 |
|  | BANK1 |
|  | CD79B |
|  | HLA-DQA1 |
|  | HLA-DQB1 |
|  | HLA-DRA |
|  | HLA-DRB1 |
|  | HLA-DPB1 |
|  | CD74 |
|  | CDKN1C |
|  | MS4A7 |
|  | SERPINA1 |
|  | IFITM3 |
|  | LST1 |
|  | FCER1G |
|  | FCGR3A |
|  | AIF1 |
|  | SAT1 |
|  | PPBP |
|  | NRGN |
|  | PF4 |
|  | GNG11 |
|  | CAVIN2 |
|  | TUBB1 |
|  | CLU |
|  | GP9 |
|  | HIST1H2AC |
|  | RGS18 |
|  | GATA2 |
|  | MALAT1 |
|  | IGKC |
|  | XIST |
|  | PIK3R1 |
|  | IKZF1 |
|  | MYH9 |
|  | GPBP1 |
|  | PCSK7 |
|  | UGP2 |
|  | MZB1 |
|  | JCHAIN |
|  | STMN1 |
|  | IGHG2 |
|  | IGHA2 |
|  | IGHG1 |
|  | IGHA1 |
|  | IGLC3 |
|  | IGLC2 |
|  | FCER1A |
|  | HLA-DMA |
|  | HLA-DPA1 |

| **CD19^-^/IgM^+^** | |
| --- | --- |
| From top to bottom | features |
|  | CCR7 |
|  | LEF1 |
|  | TCF7 |
|  | IL7R |
|  | TRABD2A |
|  | RGS10 |
|  | NOSIP |
|  | PIK3IP1 |
|  | RCAN3 |
|  | MAL |
|  | CCL5 |
|  | GZMA |
|  | CST7 |
|  | KLRG1 |
|  | IL32 |
|  | NKG7 |
|  | GZMH |
|  | TRGC2 |
|  | DUSP2 |
|  | GZMK |
|  | TCL1A |
|  | IGHD |
|  | CD79A |
|  | MS4A1 |
|  | CD79B |
|  | HLA-DRB1 |
|  | LHDB |
|  | AQP3 |
|  | TRAC |
|  | LTB |
|  | GPR183 |
|  | TRAT1 |
|  | FYB1 |
|  | JUNB |
|  | KLRF1 |
|  | TRDC |
|  | GZMB |
|  | KLRD1 |
|  | PRF1 |
|  | GNLT |
|  | FGFBP2 |
|  | MYOM2 |
|  | FCN1 |
|  | VCAN |
|  | MNDA |
|  | CST3 |
|  | FOS |
|  | LYZ |
|  | S100A9 |
|  | CSTS |
|  | S100A8 |
|  | NEAT1 |
|  | BANK1 |
|  | HLA-DQA1 |
|  | HLA-DQB1 |
|  | HLA-DPA1 |
|  | LINC00926 |
|  | BCL11A |
|  | STX7 |
|  | CDKN1C |
|  | MS4A7 |
|  | SERPINA1 |
|  | IFITM3 |
|  | FCGR3A |
|  | FCER1G |
|  | LST1 |
|  | AIF1 |
|  | SAT1 |
|  | TNFRSF17 |
|  | MZB1 |
|  | JCHAIN |
|  | PPIB |
|  | HSP90B1 |
|  | IGHA1 |
|  | IGHA2 |
|  | IGLC3 |
|  | IGKC |
|  | IGLC2 |
|  | CAVIN2 |
|  | GNG11 |
|  | TUBB1 |
|  | ACRB |
|  | CLU |
|  | PF4 |
|  | PPBP |
|  | NRGN |
|  | RGS18 |
|  | HIST1H2AC |
|  | LILRA4 |
|  | PLD4 |
|  | UGCG |
|  | PTGDS |
|  | IRF8 |
|  | CCDC50 |
|  | IRF8 |
|  | CCDC50 |
|  | TCF4 |
|  | IRF7 |
|  | PPP1R14B |

| **CD19^+^/IgM^+^** | |
| --- | --- |
| From top to bottom | features |
|  | TCL1A |
|  | IGHD |
|  | CXCR4 |
|  | HVCN1 |
|  | FECER2 |
|  | IL4R |
|  | MEF2C |
|  | LINC02397 |
|  | PLPP5 |
|  | YBX3 |
|  | GIMAP7 |
|  | LIL32 |
|  | CD3E |
|  | CD3D |
|  | CTSW |
|  | TRAC |
|  | IL7R |
|  | CCL5 |
|  | NKG7 |
|  | GNLY |
|  | MARCKS |
|  | TNFRSF13B |
|  | CD1C |
|  | MS4A1 |
|  | CCDC50 |
|  | COTL1 |
|  | CLECL1 |
|  | SPIB |
|  | GPR183 |
|  | CRIP1 |
|  | TNFRSF17 |
|  | MZB1 |
|  | JCHAIN |
|  | HSP90B1 |
|  | PPIB |
|  | IGHA1 |
|  | IGHA2 |
|  | IGHG1 |
|  | IGHG2 |
|  | IGKC |

| **Supplementary Table 5 The different distribution of CD19^-^/IgM^-^, CD19^-^/IgM^+^, CD19^+^/IgM^+^ and CD19^+^/IgM^-^ in MCL, CLL and healthy donor cells.** | | | | |
| --- | --- | --- | --- | --- |
| **Cells** | **CD19^–^/IgM^–^(%)** | **CD19^–^/IgM^+^(%)** | **CD19^+^/IgM^+^(%)** | **CD19^+^/IgM^–^(%)** |
| **JeKo-1-LZ1^*^** | **10.20** | **69.64** | **19.47** | **0.69** |
| **JeKo-1-spheroid^*^** | **10.57** | **85.07** | **0.32** | **4.03** |
| **Normal 1^a#^** | **97.53** | **2.06** | **0.18** | **0.23** |
| **Normal 2^b#^** | **82.52** | **16.83** | **0.64** | **0.02** |
| **Normal 3^b#^** | **54.39** | **42.84** | **2.72** | **0.05** |
| **Pt1^b#^** | **99.15** | **0.67** | **0.07** | **0.11** |
| **Pt2^a#^** | **98.91** | **0.76** | **0.20** | **0.13** |
| **Pt3^a#^** | **98.88** | **0.91** | **0.12** | **0.09** |
| **Pt4^c#^** | **77.89** | **22.02** | **0.05** | **0.04** |
| **Pt5^a#^** | **99.12** | **0.18** | **0.17** | **0.53** |
| **Pt6^b#^** | **99.58** | **0.05** | **0.13** | **0.24** |
| **Pt7^b#^** | **77.11** | **21.86** | **0.92** | **0.10** |
| **Pt8^a*^** | **69.64** | **2.05** | **0.21** | **28.10** |
| **Pt9^a*^** | **51.20** | **36.20** | **1.96** | **10.65** |

^*a^:BMMCs

^b^:PBMCs

^c^:MCs from Carcinomatous Hydrothorax.

^#^:The data were obtained by FCM.

^*^:The data were obtained by scRNA-seq.

**Supplementary Table 6. Analysis of tumor xenograft induction in NOD/SCID mice by pt1-derived sub-clones.**

| **Cell** | **Dose** | **# of mice with tumours** | **# of tumor-infiltrated organs** | **# of infected organs** | **# of hemorrhagic organs** |
| --- | --- | --- | --- | --- | --- |
| CD19^–^/IgM^–^ 100 4/4 4/44 1/44 1/44  10000 4/4 7/44 4/44 2/44  CD19^–^/IgM^+^ 100 4/4 4/44 0/44 0/44  10000 4/4 4/44 4/44 3/44  CD19^+^/IgM^+^ 100 4/4 4/44 2/44 0/44  10000 4/4 4/44 2/44 2/44  CD19^+^/IgM^-^  100 4/4 4/44 0/44 0/44  10000 4/4 4/44 0/44 0/44 | | | | | |
